# Evaluation of Transient Elastography, Acoustic Radiation Force Impulse Imaging (ARFI), and Enhanced Liver Function (ELF) Score for Detection of Fibrosis in Morbidly Obese Patients

**DOI:** 10.1371/journal.pone.0141649

**Published:** 2015-11-03

**Authors:** Thomas Karlas, Arne Dietrich, Veronica Peter, Christian Wittekind, Ralf Lichtinghagen, Nikita Garnov, Nicolas Linder, Alexander Schaudinn, Harald Busse, Christiane Prettin, Volker Keim, Michael Tröltzsch, Tatjana Schütz, Johannes Wiegand

**Affiliations:** 1 IFB AdiposityDiseases, University of Leipzig, Leipzig, Germany; 2 Department of Medicine, Neurology and Dermatology, Division of Gastroenterology and Rheumatology, University Hospital Leipzig, Leipzig, Germany; 3 Department of Visceral, Transplantation, Thoracic and Vascular Surgery, University Hospital Leipzig, Leipzig, Germany; 4 IFB AdiposityDiseases, Research Area Bariatric Surgery, University of Leipzig, Leipzig, Germany; 5 Institute of Pathology, University Hospital Leipzig, Leipzig, Germany; 6 Institute of Clinical Chemistry, Hannover Medical School, Hannover, Germany; 7 Department of Diagnostic and Interventional Radiology, University Hospital Leipzig, Leipzig, Germany; 8 Coordination Center for Clinical Trials, University of Leipzig, Leipzig, Germany; Yonsei University College of Medicine, REPUBLIC OF KOREA

## Abstract

**Background:**

Liver fibrosis induced by non-alcoholic fatty liver disease causes peri-interventional complications in morbidly obese patients. We determined the performance of transient elastography (TE), acoustic radiation force impulse (ARFI) imaging, and enhanced liver fibrosis (ELF) score for fibrosis detection in bariatric patients.

**Patients and Methods:**

41 patients (median BMI 47 kg/m^2^) underwent 14-day low-energy diets to improve conditions prior to bariatric surgery (day 0). TE (M and XL probe), ARFI, and ELF score were performed on days -15 and -1 and compared with intraoperative liver biopsies (NAS staging).

**Results:**

Valid TE and ARFI results at day -15 and -1 were obtained in 49%/88% and 51%/90% of cases, respectively. High skin-to-liver-capsule distances correlated with invalid TE measurements. Fibrosis of liver biopsies was staged as F1 and F3 in n = 40 and n = 1 individuals. However, variations (median/range at d-15/-1) of TE (4.6/2.6–75 and 6.7/2.9–21.3 kPa) and ARFI (2.1/0.7–3.7 and 2.0/0.7–3.8 m/s) were high and associated with overestimation of fibrosis. The ELF score correctly classified 87.5% of patients.

**Conclusion:**

In bariatric patients, performance of TE and ARFI was poor and did not improve after weight loss. The ELF score correctly classified the majority of cases and should be further evaluated.

## Introduction

Morbid obesity has a growing prevalence worldwide and affects both quality of life and life expectancy. Obesity is strongly associated with non-alcoholic fatty liver disease (NAFLD) and its sequelae, which contribute to the increased morbidity and mortality in affected patients [1]. Among different treatment approaches for higher grades of obesity, bariatric surgery is the most efficient method for weight reduction and relief of hepatic fat load [2]. However, advanced liver injury is a major risk factor for complications in bariatric surgery and should therefore be excluded prior to bariatric interventions [2, 3]. Liver histology is the current reference standard for grading and staging of fatty liver disease [4], however, its clinical implementation in the evaluation of candidates for bariatric surgery is impaired by procedure associated risks and sampling errors [1, 5].

Assessment of liver stiffness as a surrogate marker for liver fibrosis is increasingly applied for non-invasive staging of patients with chronic liver diseases [6]. Transient elastography (TE) and acoustic radiation force impulse imaging (ARFI) show high accuracy for detection of advanced liver fibrosis and cirrhosis [6–10]. The development of a special transducer for obese patients (XL probe for TE) has increased the method’s applicability in obese patients [11–13], and recent data promote the use of TE for characterization of liver fibrosis in candidates for bariatric surgery, who are at risk for advanced stages of NAFLD [14]. In this line, serum-based fibrosis scores represent a further alternative to liver histology [15, 16] and may be combined with elastography-based methods [17], but require validation in bariatric patients.

We performed a head to head comparison of ARFI, TE, and enhanced liver function (ELF) score in patients scheduled for bariatric surgery before and after a pre-interventional hypocaloric diet meant to reduce liver volume.

## Patients and Methods

### Ethical approval and informed consent

The study was performed in accordance with the ethical standards of the 1964 Helsinki Declaration and its later amendments. The study was approved by the local ethics committee (University of Leipzig, register no. 205-12-02072012). All participants provided written informed consent.

### Study cohort

Patients with indication for bariatric surgery due to (i) morbid obesity defined by BMI > 40kg/m^2^ or (ii) advanced obesity (BMI > 35 kg/m^2^) and secondary complications (type 2 diabetes mellitus, hypertension, dyslipidemia, obstructive sleep apnea syndrome) with insufficient response to a conventional multimodal conservative weight reduction therapy were recruited for a randomized controlled trial (BARO-DIET study, UTN U1111-1119-0341), which randomly investigated the effect of two different 14-day low energy diets on changes in liver volume and liver fat content prior to bariatric surgery. Exclusion criteria comprised age <18 years, pregnancy, alcohol consumption > 20 g/d, systemic steroid therapy, and severe chronic systemic diseases including malignancy.

For the present study, a subgroup of subsequent BARO-DIET patients were recruited for additional ultrasound examinations between January 2012 and April 2013. Study examinations were performed on days -15 and -1 prior to bariatric surgery and compared with intraoperative liver biopsies. In addition, a cohort of non-bariatric NAFLD patients served as reference for TE, ARFI, and ELF score applicability and diagnostic performance [18].

### Liver histology

Liver tissue samples were gained by intraoperative wedge biopsies during the bariatric intervention from all study participants. Diagnosis of NAFLD or NASH and determination of the NAFLD activity score (NAS) were performed by a single expert pathologist (CW) blinded to the clinical data as described before [18]. Steatosis and fibrosis were classified according the NAS staging [19, 20].

### Magnetic resonance spectroscopy and volumetry

MR examinations were performed on a 1.5-T scanner (Achieva XR, Philips Healthcare, Best, Netherlands) as previously described in detail before [18]. In brief, single-voxel MR spectra were acquired using a point-resolved spectroscopy (PRESS) technique. Voxels sized 8 cm^3^ were placed in liver segment VII avoiding larger bile ducts and vessels. MR spectra were analyzed with a commercial tool that determines the relative concentrations of hepatic lipids (LCModel 6.3, Oakville, Canada) and the liver fat content was calculated as relative hepatic fat fraction (given in %). The liver volume calculation was performed using a custom-made software tool (Matlab, MathWorks, Natick, MA, USA).

### Ultrasound and elastography examinations

For assessment, patients were examined in a supine position with the right arm elevated above the head. Skin-to-liver-capsule-distance (SLD) on the measuring site was determined using a 10 MHz linear ultrasound transducer, and mechanical cholestasis was ruled out in all subjects by conventional ultrasound (4 MHz transducer). Liver stiffness measurement (LSM) was performed with transient elastography (TE; Fibroscan, Echosens, Paris, France) with either M- (in cases with SLD <25 mm) or XL-probes (for cases with SLD ≥25 mm) according to the manufacturers recommendations. Examinations with less than 10 valid measurements or an interquartile range (IQR) >30% of the median LSM value (only in cases with liver stiffness ≥7.1 kPa) were excluded from further analysis [21].

ARFI examinations (Acuson S2000; Siemens Medical Solutions, Mountain View, California, USA; Software Version 350.3.044.36; convex probe 4C1, Siemens Healthcare) were performed by experienced examiners (TK, VK, MT, and JW) in the right liver lobe through the intercostal space in the same region of TE measurements as described previously [22]. In brief, the ARFI measurement box was set to an area without visible bile ducts and vessels in the B-mode image, and subsequent measurements were performed. The measurement depth from the liver capsule was between 20 and 80 mm at a region with liver parenchyma >6 cm. The shear-wave velocity was indicated in m/s and the median value of 10 valid single shots was used for further analysis. For valid ARFI results, a success rate of >60% was required. In addition, the IQR was calculated in analogy to the TE examination.

### Laboratory assessment and ELF score

Blood samples were collected from all patients at both study time points. Routine liver function tests were performed immediately and patients with elevation of aminotransferases (ALT and AST) >5 times the upper limit of normal were excluded from the further examination.

For the ELF score, stored (-20°C) serum samples were analyzed in October 2013 according to the manufacturer’s instructions as described in [16]. In brief, the ELF score combines quantitative serum concentration measurements of three fibrosis markers (tissue inhibitor of metallo-proteinases-1 –TIMP-1, amino-terminal propeptide of type III procollagen–PIIINP, and hyaluronic acid–HA) to a single value. The ELF score was calculated by the following equation: *ELF score* = 2.494 + ln(*C*_*HA*_) + ln(*C*_*PIIINP*_) + ln(*C*_*TIMP*−1_)

### Non-invasive assessment of fibrosis risk in bariatric patients

Previously published cut-off values for TE and ARFI were applied for non-invasive definition of fibrosis risk. According to a recent histology controlled study in bariatric patients, subjects with LSM >7.6 kPa were classified at risk for significant liver fibrosis or cirrhosis [14]. For ARFI, no specific cut-off values have been established in the bariatric setting so far. We therefore decided to use a cut-off value of 1.35 m/s to identify patients at risk for significant liver fibrosis or cirrhosis, which corresponds to results of two meta-analyses on ARFI accuracy [8, 9].

For the ELF score, few published data on non-invasive detection of NAFLD related fibrosis are available. Moreover, published cut-off values vary according to the applied ELF algorithm [16, 23, 24]. We therefore decided to evaluate the ELF score in a non-bariatric cohort consisting of n = 48 patients with biopsy-proven NAFLD in order to establish a cut-off value for detection of significant (≥F2) liver fibrosis. Clinical characteristics of this cohort have been previously reported in detail [18].

### Statistical analysis

Ordinal and nominal data were collected in a Microsoft® Excel file. Statistical analyses were conducted by using MedCalc® 14.12 (MedCalc Software, Belgium). Data were expressed as median and range. Fisher’s exact test and chi-square tests were used to test for the association of variables. Nonparametric tests were chosen to compare median values of two independent samples (Mann-Whitney U test) or groups (Kruskal-Wallis test) as well as paired samples (Wilcoxon test). P-values <0.05 indicated a significant difference. For correlation analysis, Spearman’s rank correlation coefficient (rho) was calculated.

TE, ARFI, and ELF score results are presented as boxplots and line charts. Diagnostic performance of the ELF score was assessed using receiver operating characteristic (ROC) curves and the area under the ROC curve (AUROC) was calculated. Optimal cut-off values were determined according to the Youden index. The probabilities of a true-positive (sensitivity, sens.) and a true-negative (specificity, spec.) were estimated as the proportions in the non-bariatric NAFLD cohort.

## Results

### Characteristics of the study cohort

**Fig 1:**
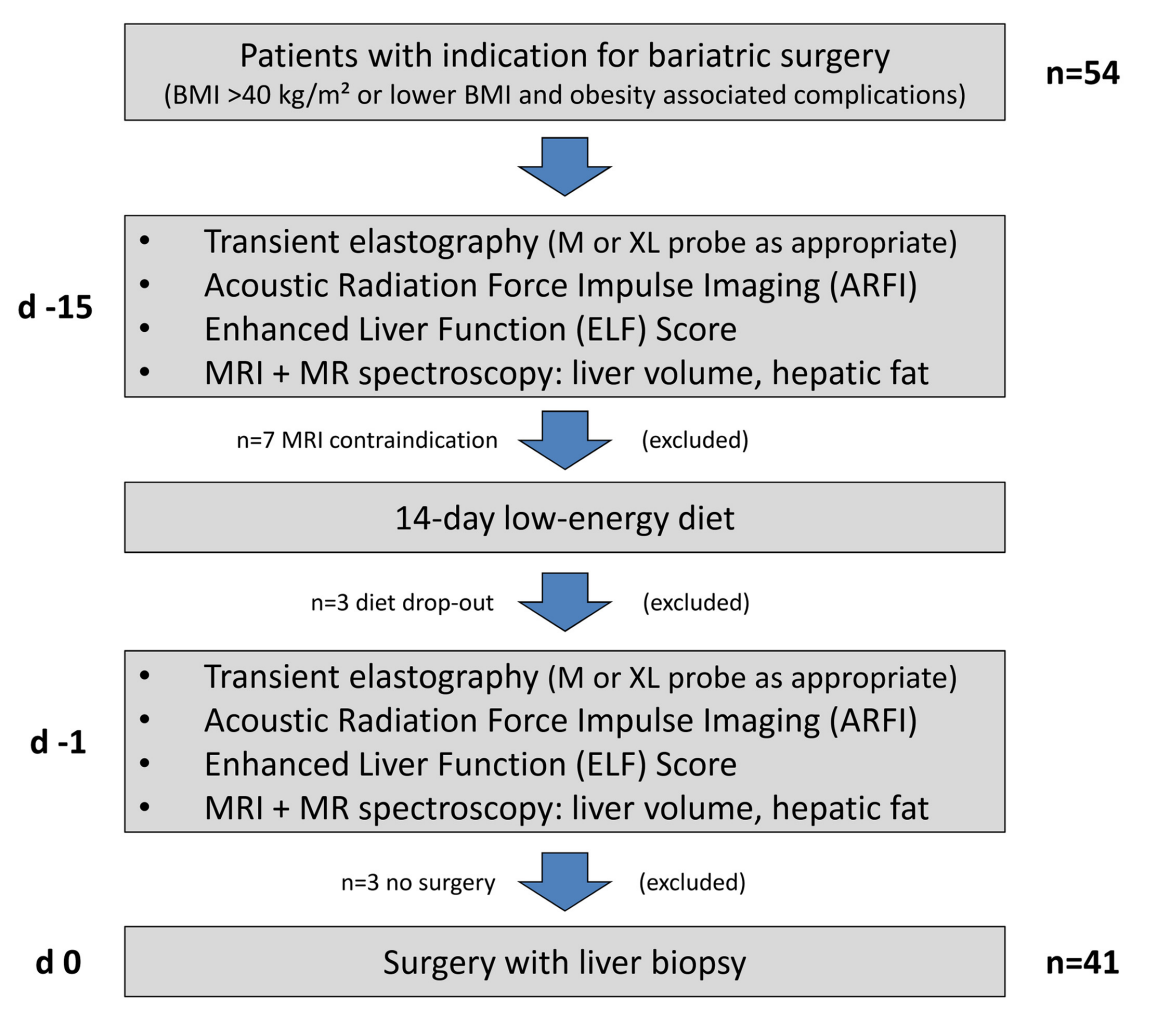
Study cohort.

54 subsequent patients from the BARO-DIET study were screened for additional ultrasound examinations. Due to contraindications for BARO-DIET study examinations or diet drop-outs 13 patients were excluded. Thus, 41 cases were considered for the final analysis (Fig 1, Table 1). The randomly assigned diets resulted in a mean BMI change of -1.79 ± 0.71 kg/m^2^ and a reduction of liver volume after 14 days of hypocaloric regimen (Table 1). This was associated with slight but significant decreases of BMI, hepatic fat content, and skin-to-liver capsule distance at the TE measuring site (Table 1).

**Table 1:** Characteristics of the study cohort.

Parameter	Bariatric patients (n = 41)		
sex (f / m)	28 / 13 (68% / 32%)		
age (years)	45.7 ± 10.2		
	*before diet*	*after diet*	*Wilcoxon test*
body mass index (kg/m^2^)	47.2 [33.7–60.1]	46.8 [32.1–57.1]	p<0.0001
• ≤ 40 kg/m^2^	2 (5%)	4 (10%)	
• > 40–45 kg/m^2^	12 (29%)	13 (32%)	
• > 45–50 kg/m^2^	12 (29%)	17 (41%)	
• > 50–55 kg/m^2^	12 (29%)	6 (15%)	
• > 55 kg/m^2^	3 (7%)	1 (2%)	
MR spectroscopy (hepatic fat fraction, %)	12.7 [0.3–34.9]	11.4 [0.5–41.4]	p = 0.0339
liver volume (ml)	2437 [1762–4308]	2226 [1433–3464]	p<0.0001
Skin-to-liver-capsule distance at TE site (mm)	39 [17–71]	34.7 [27–52]	p = 0.0117
• < 25 mm	1 (2%)	0 (0%)	
• 25–35 mm	14 (34%)	22 (54%)	
• > 35 mm	26 (63%)	19 (46%)	
	***Liver histology***		
*Fibrosis*			
• F0-1	40 (98%)		
• F2-4	1 (2%)		
*Steatosis*			
• S1	25 (61%)		
• S2	10 (24%)		
• S3	6 (15%)		
*NASH (NAS)*			
• NAS ≤4	22 (54%)		
• NAS >4	19 (46%)		

TE—transient elastography; NASH–non-alcoholic steatohepatitis; NAS–NAFLD activity score

### Applicability and diagnostic accuracy of non-invasive methods in the bariatric patient cohort

The use of TE M probe was indicated in only one patient (Table 1), for whom no valid LSM could be obtained with either probe. Thus, all valid TE measurements were performed with the XL probe. Compared with ARFI, TE showed a significantly reduced applicability at both study examinations (p<0.001, respectively; Table 2). TE failure (both time points) was associated with SLD > 35 mm (62 vs. 34%, p = 0.0236). However, the measuring range of individual ARFI results was high and the IQR was > 30% of the median value in 70% of cases with successful measurements.

**Table 2:** Applicability and diagnostic value of non-invasive methods for fibrosis detection prior to bariatric surgery.

**Method applicability**		**TE** ^**a**^	**ARFI**	**ELF score**
Cases with valid results	before diet	20 (49%)	36 (88%)	40 (100%)^b^
	after diet	21 (51%)	37 (90%)	39 (100%)^b^
	serial measurements	15 (37%)	34 (83%)	38 (100%)^b^
**Performance for fibrosis detection**		**TE**	**ARFI**	**ELF score**
*cut-off value*		*7*.*6 kPa*	*1*.*35 m/s*	*9*.*92*
correctly classified cases (≤F1)	before diet	15 (79%)	12 (34%)	34 (87%)
	after diet	12 (57%)	12 (33%)	33 (87%)
F3 case correctly classified?	before diet	+	+	+
	after diet	invalid	+	+

The category “correctly classified” indicates the proportion of F0-1 cases with liver stiffness measurement results below the respective cut-off values for significant fibrosis (≥F2).

TE—transient elastography; ARFI–Acoustic Radiation Force Impulse Imaging

^a^ According to the manufacturer’s recommendation, the use of XL probe was indicated in all cases with valid measurements (skin-to-liver-capsule distance > 25 mm).

^b^ ELF score could be calculated from all analyzed serum specimens. However, serum samples for ELF score were not available in one (before diet) and two (after diet) cases, respectively.

Quantitative serum concentration measurements of the ELF score fibrosis markers could be successfully performed from all stored serum samples (Table 2). Blood samples for ELF score testing were not available in one (before diet) and two (after diet) patients (all histologically classified as F1 fibrosis) for logistic reasons.

### Applicability and diagnostic accuracy for non-invasive estimation of liver fibrosis in the non-bariatric control group

The diagnostic performance of TE, ARFI, and the ELF score for detection of significant liver fibrosis (≥F2) was evaluated in n = 48 patients of a non-bariatric cohort (n = 24 male; age 55.3±11.0 years; BMI 27.5 ± 4.3 kg/m^2^; n = 8 with fibrosis ≥F2). Detailed patient characteristics of this cohort have been reported before [18]. However, two individuals of this study were excluded from the current analysis because they underwent bariatric treatment in their further therapeutic concept.

TE was feasible in n = 45 (94%) patients, the XL probe was used in n = 13 (29%) cases. Using the probe algorithm as recommended by the manufacturer TE correctly identified all patients with liver fibrosis ≥F2 (sensitivity 100%, specificity 100%, AUROC 1.0, cut-off 8.05 kPa). ARFI was feasible in all patients. At a cut-off value of 1.245 m/s, ARFI achieved a high diagnostic accuracy for detection of liver fibrosis ≥F2 (sensitivity 100%, specificity 82.5%, AUROC 0.959). Analysis of ELF score results revealed an AUROC of 0.953 at a cut-off point of 9.92 (≥F2: sensitivity 100%, specificity 90%).

### Diagnostic accuracy for non-invasive estimation of liver fibrosis in bariatric patients

Intra-operative wedge biopsies (> 50 portal tracts) could be obtained in all patients during the bariatric intervention and served as reference standard for the non-invasive methods. Histology revealed high prevalences of steatosis (present in all patients, 39% with advanced steatosis ≥S2) and NASH (46%), but only one case (2%) with advanced F3 liver fibrosis (Table 1).

**Fig 2:**
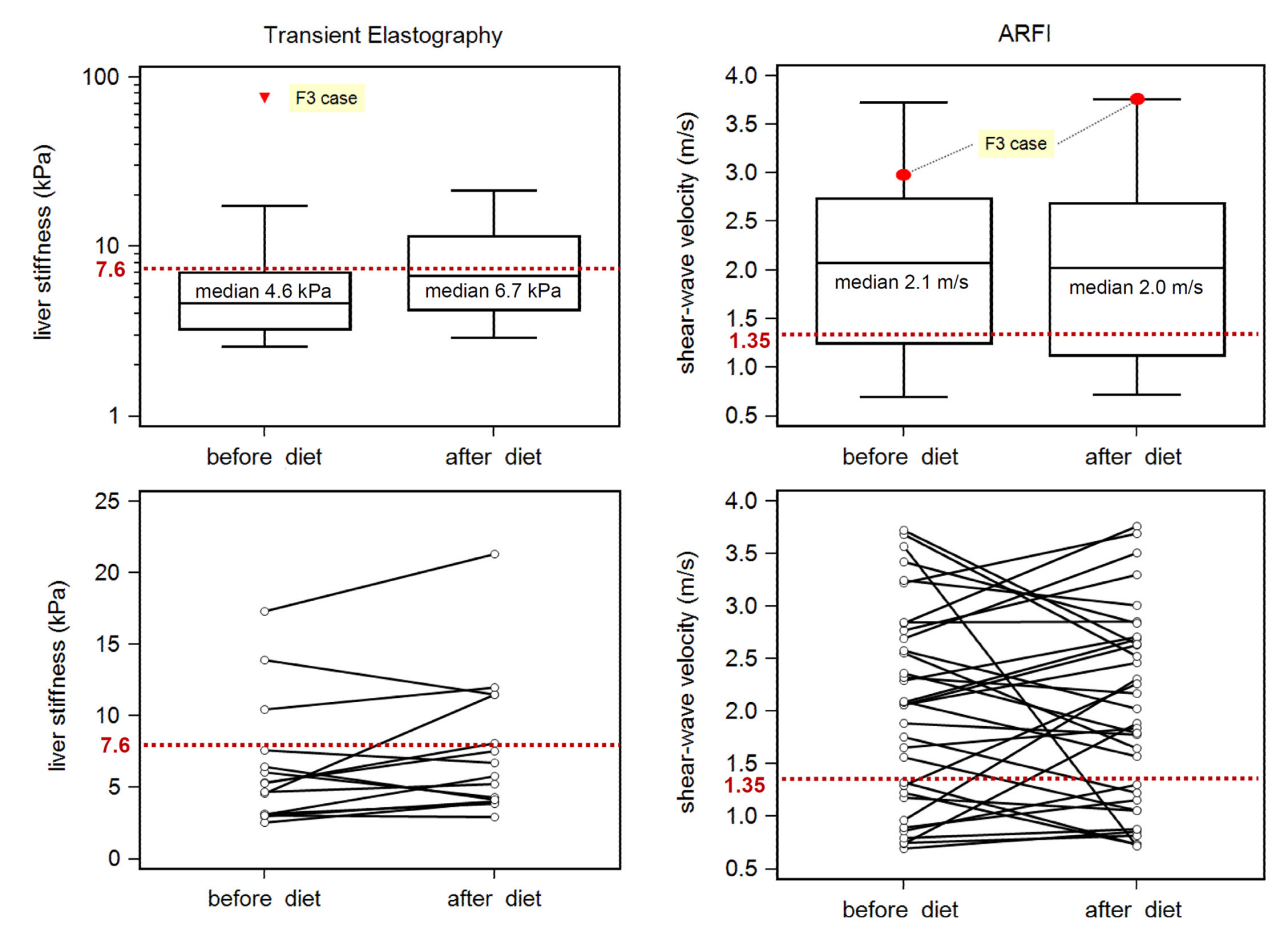
Liver elastography in patients scheduled for bariatric surgery. Red lines indicate recommend cut-offs for detections of advanced liver fibrosis (TE) [13] and liver cirrhosis (acoustic radiation force impulse imaging, ARFI) [8]. Application of these cut-offs in patients scheduled for bariatric surgery results in overestimation of fibrosis severity in a high percentage of cases.

In contrast to the biopsy proven fibrosis results, variations of TE and ARFI measurements were high (median/range before and after diet): 4.6 (2.6–75) and 6.7 (2.9–21.3) kPa for TE and 2.1 (0.7–3.7) and 2.0 (0.7–3.8) m/s for ARFI, respectively (Table 2, Fig 2). Compared to TE, ARFI showed a higher rate of incorrectly classified cases before diet (p = 0.0038; Table 2). No significant changes were observed in patients with available serial measurements before and after diet (p>0.15 for ARFI and TE, respectively). In patients without advanced fibrosis (n = 40), higher median LSM values were observed in cases with SLD > 35 mm at both examinations before and after dietary intervention: TE 7.0 vs. 3.4 kPa (p = 0.003) and 7.8 vs. 5.0 kPa (p = 0.154), and ARFI 2.4 vs. 1.1 m/s (p<0.001) and 2.6 vs. 1.3 m/s, respectively.

Because TE failure was associated with high SLD values, we performed an analysis of the impact of SLD and BMI on the applicability and diagnostic accuracy of ARFI and TE. For cases without advanced fibrosis (n = 40), valid and accurate TE measurements could be optimally discriminated by SLD values of ≤ 34.8 mm (before diet; sens. 73% and spec. 88%) and ≤ 39.0 mm (after diet; sens. 92% and spec. 43%), respectively. This was associated with BMI values ≤ 49.3 kg/m^2^ (before diet: sens. 100% and spec. 64%) and ≤ 47.2 kg/m^2^ (after diet: sens. 92% and spec. 57%), respectively. In this line, accurate ARFI measurements were associated with SLD values of ≤ 35.8 mm (before diet: sens. 83% and spec. 79%) and ≤ 33.3 mm (after diet: sens. 83% and spec. 75%) and BMI values of > 48.1 kg/m^2^ (before diet: sens. 57%, spec. 83%) and > 47.3 kg/m^2^ (after diet: sens. 50%, spec. 83%), respectively.

**Fig 3:**
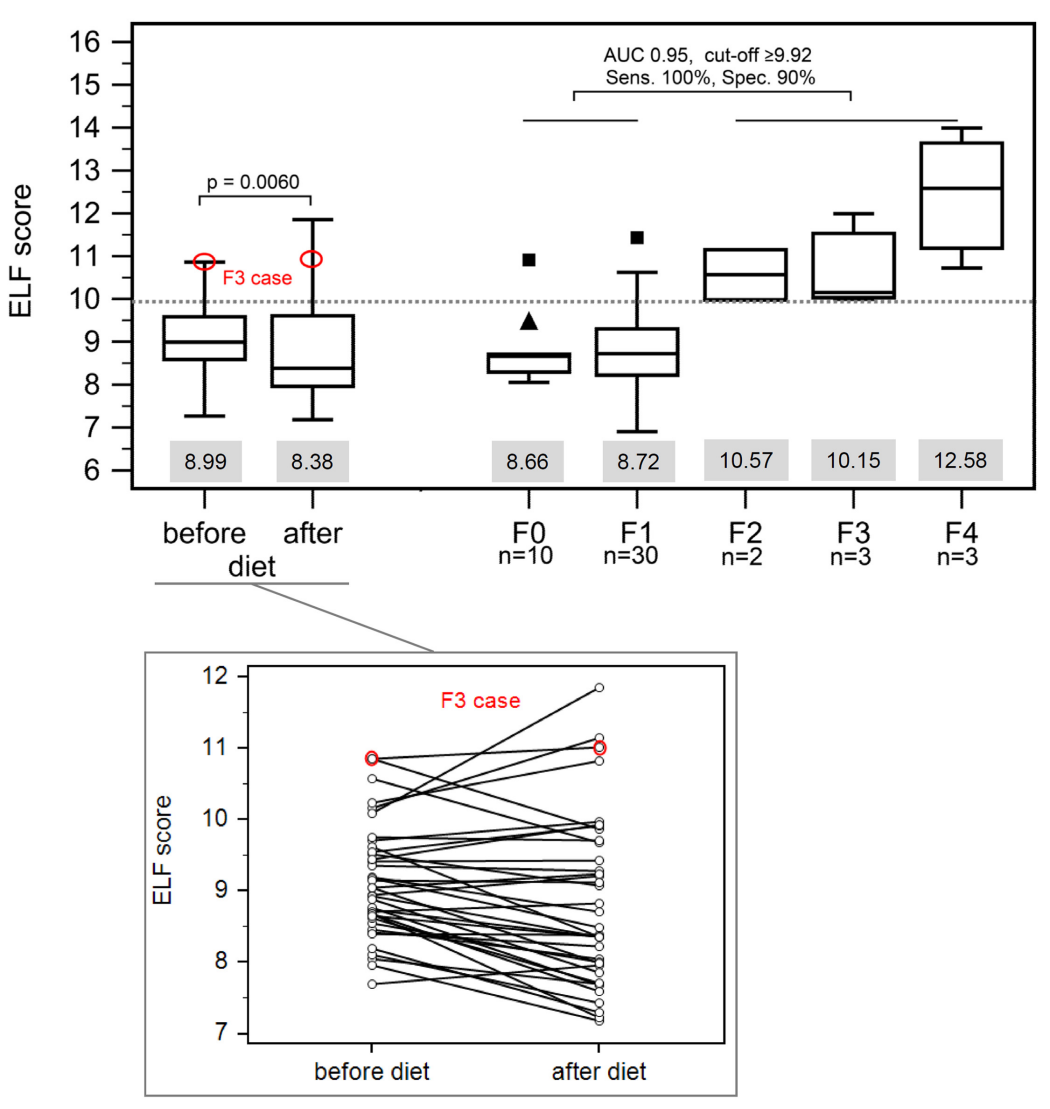
Performance of ELF score. The diagnostic accuracy of the ELF score for detection of advanced liver fibrosis (≥F2) was evaluated in a cohort of non-bariatric NAFLD patients [17]. The calculated cut-off was applied to the bariatric cohort and correctly classified the majority of patients. Grey boxes below each bar indicate the median value.

In contrast to TE and ARFI, the ELF score had a higher specificity for exclusion of fibrosis ≥F2. Applying the cut-off value of 9.92 from the non-bariatric control group in the bariatric cohort, the ELF score correctly classified 87.5% of cases including the patient with advanced fibrosis with higher variation after dietary intervention: 9.0 [7.3–10.9] and 8.4 [7.2–11.9] (p = 0.009) (Table 2, Fig 3).

### Impact of steatosis and steatohepatitis on TE, ARFI, and ELF score

TE, ARFI, and ELF score values did not show a significant correlation with the histologically determined degree of hepatic steatosis before and after diet according to Spearman’s rank correlation analysis. However, liver stiffness assessed by TE was marginally increased in cases with advanced steatosis (S2-3) before diet (5.7 vs. 3.9 kPa, p = 0.05), while no such effect was observed by TE after diet as well as for ARFI and ELF at both time points.

To evaluate the influence of hepatic inflammatory activity on the methods’ diagnostic performance, we compared test results of patients with and without severe steatohepatitis. Patients with significant hepatocellular inflammation (NAS > 4) had higher median TE (before diet: 6.8 vs. 3.8 kPa, p = 0.011; after diet: 7.8 vs. 4.1 kPa, p = 0.043) and ARFI values (before diet: 2.47 vs. 1.31 m/s, p = 0.011; after diet: 2.40 vs. 1.65 m/s, p = 0.068) compared to cases with lower inflammatory activity (NAS ≤ 4). No impact of steatohepatitis severity was observed on ELF score results (before diet: 9.12 vs. 8.91, p = 0.644; after diet: 8.38 vs. 8.53, p = 0.876).

## Discussion

Pre-operative characterization of liver fibrosis is of major importance in patients scheduled for bariatric surgery, because it contributes to the peri-interventional risk assessment [2, 3] and helps to better understand the burden of liver disease in bariatric patients [25]. Liver biopsy is considered the gold standard for staging and grading of non-alcoholic fatty liver disease, however, it is mostly not feasible due to procedure associated risks and sampling errors in morbid adiposity [1, 7]. Alternatively, non-invasive ultrasound techniques may be applied, which, however, are also limited by anthropometry of severely obese patients [1, 26, 27]. Our study represents the first head-to-head comparison of different modern non-invasive approaches for pre-interventional NAFLD staging in bariatric patients. In contrast to many non-bariatric cohorts and our own control group, where TE and ARFI achieved high diagnostic accuracies for detection of advanced liver fibrosis when either applied alone [6–10, 18, 26], or in combination with serum fibrosis markers [15, 17], we observed a wide variation of LSM values resulting in an overestimation of liver fibrosis in the majority of patients. This was associated with severe limitations of applicability and reproducibility (TE) and disputable validity due to high ranges of individual single LSM (ARFI). Particularly, ARFI overestimated the severity of fibrosis in the majority of patients and thus did not provide any diagnostic benefit. Even the use of the established cut-off value for detection of cirrhosis (1.80 m/s [8]) would have resulted in an incorrect fibrosis staging in more than 50% of patients. Although our histological reference standard consisted of wedge biopsies our study clearly demonstrates the limitations of elastography-based approaches in patients with morbid obesity against the background of a low prevalence of advanced fibrosis.

Our TE data are contrary to a recent study from Naveau et al. [14] who reported a good applicability and a high negative predictive value of TE for exclusion of advanced fibrosis in a cohort of bariatric patients. However, this study recruited patients with lower BMI (mean 42.3 kg/m^2^) and lower prevalence of NASH (33%) than we observed in our study cohort. Obesity associated anthropometric factors, especially the proportion of patients with a SLD >35 mm, have a high impact on applicability and accuracy of TE, because the XL probe acquires LSM data from measurement depths of 35 to 75 mm [11]. Short-term dietary interventions do not seem suitable to improve TE performance: although the hypocaloric regimens caused significant weight loss and associated modulation of anthropometric parameters, the conditions for ultrasound-based approaches were only slightly better after diet, which resulted in persistently unfavorable examination conditions. Our data underscore that high BMI and SLD values impede ultrasound-based diagnostic approaches. Thus, liver stiffness measurement results of morbidly obese patients require careful interpretation even if recommended measurement quality criteria are fulfilled. In addition, NASH activity can alter liver stiffness and thus impairs accuracy of non-invasive fibrosis estimation [28]. Likewise, ARFI results can be modulated by measuring depth [29] and NASH activity [30]. This may also explain our observation of high individual ARFI IQR values, which are potentially associated with imprecise measurement results [31].

In contrast to elastography-based methods, the ELF score correctly classified fibrosis in the majority of patients although it must be acknowledged that the cut-off for significant fibrosis was transferred from a non-bariatric cohort and has not been formally proven in bariatric patients yet. Our ELF data are in line with data from patients with viral hepatitis, where the ELF algorithm reliably detected advanced liver fibrosis applying a similar cut-off value (9.8) [32]. This finding indicates that the ELF score is less influenced by obesity than liver elastography. However, we observed decreasing ELF score values after diet in the majority of patients which might be caused by effects of the diet regimen on ELF score variables (HA, PIIINP, TIMP-1). Pre-interventional hypocaloric regimens are nowadays implemented in the routine management of bariatric patients [33] and should therefore be considered as a confounding factor on non-invasive approaches for characterization of liver disease. In addition, ELF score modulation by age and hepatic inflammatory activity has also been reported [32]. Thus, the ELF score requires further evaluation, especially in the clinical context of obesity related NAFLD and NASH, before implementation in clinical practice can be recommended.

Finally, the add-on design of this study to a randomized-controlled trial did not allow a power calculation in regard of the prevalence of advanced liver fibrosis, which was low compared to reports from other bariatric cohorts [34] and hence prevented the calculation of cut-off values for TE and ARFI. Nevertheless, we believe that our data provide valuable information on the performance of non-invasive methods for the detection of liver fibrosis in a bariatric setting. They correspond to a recent report of a large cohort of diabetic patients, which described limited value of non-invasive markers within low disease prevalence settings [35]. This finding puts the clinical importance of fibrosis screening in such patient cohorts into new perspective und underlines the need for non-invasive methods with high specificity.

In conclusion, applicability and accuracy of TE and ARFI are limited in patients with morbid obesity. Considering the low prevalence of advanced liver fibrosis in our cohort, the high rate of false-positive elastography measurements demonstrates the technical limits of non-invasive liver evaluation in morbidly obese subjects. The ELF score correctly classified the majority of patients, but further research is required before biomarker-based fibrosis detection can be implemented in the clinical management of bariatric patients.

## Supporting Information

S1 Dataset The study data base is available for download. (XLSX)
